# Identification and Characterization of the WOX Family Genes in Five *Solanaceae* Species Reveal Their Conserved Roles in Peptide Signaling

**DOI:** 10.3390/genes9050260

**Published:** 2018-05-17

**Authors:** Xiaoxu Li, Madiha Hamyat, Cheng Liu, Ahmad Salman, Xiaoming Gao, Cun Guo, Yuanying Wang, Yongfeng Guo

**Affiliations:** Key Laboratory for Tobacco Gene Resources, Tobacco Research Institute, Chinese Academy of Agricultural Sciences, Qingdao 266101, China; liwei06@caas.cn (X.L.); star_143_101@hotmail.com (M.H.); lc4115225111@163.com (C.L.); safitfa@yahoo.com (A.S.); gaoxiaoming@caas.cn (X.G.); guocun_zzu@163.com (C.G.)

**Keywords:** WOX family, *Solanum lycopersicum*, *Solanum tuberosum*, *Nicotiana tabacum*, *Nicotiana tomentosiformis*, *Nicotiana sylvestris*, WUS box

## Abstract

Members of the plant-specific WOX (WUSCHEL-related homeobox) transcription factor family have been reported to play important roles in peptide signaling that regulates stem cell maintenance and cell fate specification in various developmental processes. Even though remarkable advances have been made in studying *WOX* genes in *Arabidopsis*, little is known about this family in *Solanaceae* species. A total of 45 WOX members from five *Solanaceae* species were identified, including eight members from *Solanum tuberosum*, eight from *Nicotiana tomentosiformis*, 10 from *Solanum lycopersicum*, 10 from *Nicotiana sylvestris* and nine from *Nicotiana tabacum*. The newly identified WOX members were classified into three clades and nine subgroups based on phylogenetic analysis using three different methods. The patterns of exon-intron structure and motif organization of the WOX proteins agreed with the phylogenetic results. Gene duplication events and ongoing evolution were revealed by additional branches on the phylogenetic tree and the presence of a partial WUS-box in some non-WUS clade members. Gene expression with or without CLE (clavata3 (clv3)/embryo surrounding region-related) peptide treatments revealed that tobacco *WOX* genes showed similar or distinct expression patterns compared with their *Arabidopsis* homologues, suggesting either functional conservation or divergence. Expression of *Nicotiana tabacum WUSCHEL* (*NtabWUS*) in the organizing center could rescue the *wus-1* mutant phenotypes in *Arabidopsis,* implying conserved roles of the *Solanaceae* WOX proteins in peptide-mediated regulation of plant development.

## 1. Introduction

The WUSCHEL-related homeobox (WOX) transcription factors belong to a plant-specific subgroup of the homeodomain (HD)-containing transcription factor superfamily, members of which possess a conserved DNA-binding homeodomain. A typical homeodomain of WOX transcription factors contains 60 amino acid residues with a helix-loop-helix-turn-helix structure [1]. Earlier expression studies and functional analyses indicated that WOX family members participate in various developmental processes of plants, including stem cell homeostasis and organ formation [1,2]. 

WOX transcription factors are broadly distributed in plant species, from ancient green algae to both monocots and dicots. In a recent study, 350 WOXs with a typical homeodomain were identified from 50 species in the plant kingdom. These members were grouped into three clades: WOXs from the ancient clade were identified in all species from algae to higher plants, the intermediate clade contained WOX proteins only from vascular plant species, and the modern clade or WUSCHEL (WUS) clade members were only identified in seed plants. The WOX family in the model eudicot plant *Arabidopsis* harbors 15 WOX members and 13 WOX transcription factors were identified on the genome of the model monocot plant rice [3]. 

A number of *WOX* genes have been reported to play a significant role in peptide signal-mediated stem cell homeostasis of different types of meristems [4]. The organizing center (OC) and the quiescent center (QC) are essential to cell fate determination in the shoot and root apical meristem respectively. *Arabidopsis thaliana* WUSCHEL (*AtWUS)* which is specifically expressed in the OC has been shown to be the key transcription factor regulating stem cell homeostasis in the shoot apical meristem (SAM) [5]. WUS functions downstream of the CLAVATA (CLV) signaling pathway in which the CLV3 peptide signal is perceived by the CLV receptors including CLV1, CLV2, BAM1/BAM2 (barely any meristem 1/barely any meristem 2), and receptor-like protein kinase 2 (RPK2) [6,7]. Type 2C protein phosphatases POLTERGEIST and POLTERGEIST LIKE1 are signaling intermediates between CLV3 perception and WUS [8,9]. Transduction of the CLV signal leads to the restriction of *WUS* expression within the OC, while on the other hand *WUS* promotes expression of the peptide-encoding *CLV3*, forming a negative feedback loop, through which the population of stem cells within the SAM is maintained at a relatively constant level [10]. At the other end of a plant, *AtWOX5* is specifically expressed within the QC in the root apical meristem (RAM) to define QC identity and regulate division and differentiation of RAM stem cells. Similar to WUS, WOX5 functions downstream of the signaling pathway initiated by another CLE peptide, CLE40 [11,12]. In fact, WOX5 can act interchangeably with WUS in the control of shoot and root stem cell niches [13,14]. Furthermore, AtWOX4 functions redundantly with AtWOX14 in defining the vascular stem cell niche together with the CLE peptide TDIF (tracheary element differentiation inhibitory factor) and its receptor TDIF receptor (TDR)/phloem intercalated with xylem (PXY) [15].

In addition, most of the other *Arabidopsis WOX* genes have also been shown to play regulatory roles in various cell fate determination processes including organogenesis and patterning. *AtWOX2* and *AtWOX8* function together in regulating apical-basal axis formation during embryogenesis [16]. *AtWOX3* is expressed in the periphery of the shoot meristem and functions during lateral organ formation [17,18]. *AtWOX6* is highly expressed in developing ovules and plays a central role in ovule patterning [19]. *AtWOX7* is involved in lateral root formation [20]. *AtWOX9* was found to functionally overlap with *AtWOX8* in promoting embryonic cell division and promoting growth of the vegetative SAM at later development stages [16,21,22]. *AtWOX11* and *AtWOX12* function redundantly in regulating the first-step cell fate transition during de novo root organogenesis of *Arabidopsis* leaf explants [23]. AtWOX13 was reported to be involved in replum development and fruit patterning [24].

Besides *Arabidopsis*, regulatory roles of *WOX* genes have been studied in a number of different plant species including rice, maize, sorghum, poplar, paper mulberry, grape, petunia, *Pisum sativum* L, *Pyrus bretschneideri, Medicago truncatula* and *Nicotiana sylvestris* [3,25,26,27,28,29,30,31]. Functions of WOX orthologues could be conserved or divergent among species [1]. However, only a limited number of *WOX* genes have been identified in the *Solanaceae* species so far and little is known about their functions [31].

In this study, we carried out a systematic study to identify and characterize the *WOX* gene family on the genomes of five *Solanaceae* species including tomato (*Solanum lycopersicum*), potato (*Solanum tuberosum*), common tobacco (*Nicotiana tabacum*), *N. sylvestris* and *Nicotiana tomentosiformis*. Among the *Nicotiana* species (collectively referred to as tobacco), common tobacco is an allotetraploid generated via natural crossing between *N. sylvestris* and *N. tomentosiformis.* We analyzed the expression patterns of *WOX* genes from *N. tabacum* and *N. sylvestris* and in comparison with their counterparts in *Arabidopsis*. Changes in expression of the *N. tabacum WOX*s after CLE peptide treatments were also determined to understand their potential roles in peptide signaling. Furthermore, we analyzed the function of NtabWUS in maintaining the shoot apical stem cell niche and in floral organ development.

## 2. Materials and Methods

### 2.1. Identification of WOX Members

The latest versions of the genome annotations of *S. lycopersicum* (ITAG, Release 2.4)*, S. tuberosum* (PGSC, Release 3.4)*, N. tabacum* (K326)*, N. sylvestris* and *N. tomentosiformis* were downloaded from SGN (Sol Genomics Network, http://solgenomics.net/). Previously reported AtWOX full-length and homeodomain amino acid sequences [3] were retrieved from The Arabidopsis Information Resource (TAIR: http://www.arabidopsis.org/), aligned with MAFFT v5.3 to produce Stockholm files and then subjected to HMMER v3.0 for building HMM (hidden Markov models) profiles. The HMM profiles were applied to perform HMM search against the annotated *Solanaceae* protein databases with an E-value cutoff of 1.0. Furthermore, using both the full-length and homeodomain amino acid sequences of AtWOXs, BLASTP search was performed to identify additional potential WOX proteins with an E-value cutoff of 0.01. The protein sequences obtained from the two above-described approaches were combined and redundant entries were removed manually. The resulted hit sequences were then analyzed with both Pfam (https://pfam.xfam.org/) and SMART (http://smart.embl.de/) to ensure the presence of the homeobox domain.

### 2.2. Multiple Sequence Alignment and Phylogenetic Analysis

Multiple sequence alignment of both full-length and homeodomain amino acid sequences of AtWOXs and putative WOX members from the *Solanaceae* species was performed using MAFFT v5.3 under the default settings with manual editing. Phylogenetic trees were constructed using three different approaches based on the alignment results. A neighbor-joining (NJ) tree was constructed from the alignment of full-length amino acid sequences of AtWOXs and *Solanaceae* WOX members using MEGA package 6.06 with the following parameters: Poisson correction, pairwise deletion and bootstrap values (1000 replicates). A maximum likelihood (ML) tree was generated from the alignment of homeodomain amino acid sequences of AtWOXs and *Solanaceae* WOX members with the PhyML software package 3.0 using 100 replicates and the JTT model with gamma-distribution of rates (JTT+G model) advised by ProtTest 2.4. A Bayesian inference (BI) tree was constructed from the alignment of homeodomain amino acid sequences of AtWOXs and *Solanaceae* WOXs. The same JTT+G model was selected with the assistance of ProtTest 2.4. For the BI tree, the analysis was performed with MrBayes (http://mrbayes.sourceforge.net/) for 2,500,000 generations with trees sampled every 1,000 generations and a burn-in of 625. Theses tree files were visualized with FigTree 1.4.0.

### 2.3. Gene Structure and Motif Analysis

Exon-intron structures were analyzed and illustrated with the Gene Structure Display Server (GSDS: http://gsds.cbi.pku.edu.cn/) [32] using both coding sequences (CDS) and genomic sequences obtained from the Sol Genomics Network. The conserved motifs in AtWOXs and *Solanaceae* WOX proteins were identified with the assistance of MEME 4.9.1 (Multiple Expectation Maximization for Motif Elicitation, http://meme-suite.org/), and visualized by WebLogo [33] (http://weblogo.berkeley.edu/logo.cgi). Parameters were set as follows: distribution of motif occurrences, zero or one per sequence; maximum number of motifs, 8; optimum motif width, ≥6 and ≤ 100; and the optimum number of sites for each motif, ≥2 and ≤200.

### 2.4. Tobacco Materials and Growth Conditions

*Nicotiana sylvestris* and *N. tabacum* (cultivar K326) seeds were sowed in soil pots covered with plastic film. Plants were grown in a growth room at 25 °C under continuous light. Seven-week-old (8 leaf stage) plants of *N. sylvestris* and *N. tabacum* were used for gene expression analysis. Five different tissue types including root, root tip, stem, shoot tip, and leaf were used for RNA extraction. Tissues were harvested, put in liquid nitrogen immediately and stored at −80 °C before RNA extraction.

### 2.5. Synthetic Peptide Application on Tobacco Seedlings

*Nicotiana tabacum* seedlings were grown on Murashige and Skoog (MS) media containing 10 μM synthetic CLV3 peptides (CLV3p: RTVPSGPDPLHH) for 21 days. Plates were incubated at 25 °C with 16 h light and 8 h dark per day. The CLV3p were ordered from Genscript Biotechnology (Beijing, China).

### 2.6. RNA Extraction and quantitative real time-PCR

Total RNA was extracted with TRIzol (Ambion, Thermo Fisher Scientific, Waltham, MA, USA) according to the manufacturer’s instructions and treated with DNase I (Thermo Fisher Scientific) to remove DNA contamination. First-strand cDNA synthesis was performed using an M-MLV (Moloney murine leukemia virus) Reverse-Transcription Kit (Invitrogen, Carlsbad, CA, USA). The cDNA was diluted 1:30 with ddH_2_O and used as templates for quantitative real time PCR (qRT-PCR). The FastStart Universal SYBR Green Master (ROX, Roche, Basel, Switzerland) was used to detect gene expression. The qRT-PCR was performed on a 7500 Real-Time PCR System (Applied Biosystems, Waltham, MA, USA) and PCR reactions were performed in a total volume of 20 µL containing 1 µM of each primer (0.6 µL), 50 ng/µL cDNA (2 µL), and 2 × FastStart Universal SYBR Green Master (10 µL). Gene-specific primers were designed using Primer 5. The *N. tabacum* and *N. sylvestris beta-actin* genes were used as internal controls. Relative gene expression levels were calculated using the 2^−ΔΔ*C*T^ method. Primers used are listed in Supplementary Table S1.

### 2.7. Expression Analysis of AtWOXs, NsylWOXs and NtabWOXs

Expression data of *AtWOXs* were obtained from the database TraVA (travadb.org). Together with qRT-PCR data of *NsylWOXs* and *NtabWOXs*, all expression data were normalized and visualized with R [34].

### 2.8. Subcellular Localization

The coding region of *NtabWUS* excluding the terminator codon was amplified and ligated into the *pEasy-Blunt* vector, and then the *NtabWUS* and green fluorescent protein (*GFP)* fragments were cloned into the *pCHF3* vector by Infusion (Invitrogen) separately, generating the *NtabWUS-GFP* fusion construction driven by the *CaMV35S* promoter. The recombinant construct and control were used for *Agrobacterium*-mediated transient expression in *Nicotiana benthamiana* [35]. Fluorescence signals were captured using a Confocal Microscope (TCS-SP8 Leica, Wetzlar, Germany) four days after infiltration. DNA dye 4,6-diamidino-2-phenylindole (DAPI) staining was used to show the position of the nucleus.

### 2.9. Transactivation Activity Assay

The coding sequence of *NtabWUS* was inserted into the EcoRI restriction sites of the *pBridge* vector with Infusion (Clontech, Palo Alto, CA, USA). This construct and control *pBridge* vector were transformed into yeast strain AH109 (Clontech). Yeast transformation were performed following to the manufacturer’s instructions (Clontech). The transformed yeast cells were incubated on synthetic dextrose (SD) medium lacking tryptophan (SD/−Trp) and SD medium lacking tryptophan supplemented with X-Gal (SD/-Trp-x-gal) plates at 30 °C for four days. The transactivation activity was evaluated based on β-galactosidase activity.

### 2.10. Genetic Complementation Analysis

The *Arabidopsis wus-1* mutant (NASC ID: CS15) and marker line J2341 (NASC ID: CS9118) were ordered from NASC (http://arabidopsis.info/). As a typical ethyl methanesulfonate induced mutant, *wus-1* changed the second exon–intron border (G to A) and resulted in a predicted translational stop a few codons later. A specific primer pair (F: GGATTTGGGTTAGTAGAAAA/R: ATCGAAGAAGTTGTAAGGTG) was designed to amplify a fragment residing this SNP and then sent for sequencing. If the mutant was heterozygous (*wus-1* +/−), PCR with this primer pair would give a double peak with ‘G/A’, and when *wus-1* was homozygous it would show a single peak of ‘A’. The *wus-1* mutant was crossed with J2341 and presence of the GAL4-GFP enhancer trap reporter J2341 in the F_1_ plants was PCR confirmed. To generate the construct for complementation analysis, the coding sequence of *NtabWUS* was cloned into the *pPZP211* vector together with the *AtWUS* promoter. The resulted construct was used to transform *wus-1+/*− J2341 plants via the floral dip method and transformants were selected on 1/2 MS medium plates containing 50 mg/mL of Kanamycin. The Confocal Microscopy (Leica, TCS-SP8) was performed to test the GFP fluorescence and 488 nm laser lines were used for excitation of GFP [14].

## 3. Results

### 3.1. Identification of WOX Transcriptional Factors

To identify WOX family genes in the *Solanaceae* species, homeodomain sequences of 15 previously identified AtWOX proteins were employed to build the HMM profile which was then used as queries to perform HMM searches against related protein databases. Furthermore, BLASTP searches were carried out to obtain more potential WOX proteins using both full-length sequences and homeodomain sequences of known AtWOX proteins as queries. Redundant sequences were removed through manual reconstruction. To further verify the reliability of these candidate sequences, Pfam and SMART analyses were carried out to ensure the presence of the homeodomain in each candidate protein.

A total of 45 WOX proteins were identified, including eight from *N. tomentosiformis*, eight from *S. tuberosum*, ten from *N. sylvestris*, ten from *S. lycopersicum*, and nine from *N. tabacum* (Supplementary Dataset S1). Considering that *N. tabacum* is an allotetraploid which was generated via natural crossing between *N. sylvestris* and *N. tomentosiformis*, the smaller size of the *N. tabacum* WOX family could be due to low sequence conservation and/or incomplete genome information of *N. tabacum*. More WOX members might be identified in the *N. tabacum* genome in future.

To better understand the orthologous relationships between AtWOXs and the newly identified *Solanaceae* WOX members, an unrooted phylogenetic tree constructed with full-length sequences of AtWOX proteins (Supplementary Dataset S2) was employed to identify potential orthologues. Based on NJ phylogenetic analysis, homologues of AtWOXs from the *Solanaceae* species were given names with prefixes indicating species name (*Sl, S. lycopersicum; St, S. tuberosum; Ntab, N. tabacum; Ntom, N. tomentosiformis; Nsyl, N. sylvestris*) and suffix indicating subgroups of the WOX family (WOX1, WOX2 etc.). Furthermore, letters a, b, c or d were added to the end of the names based on phylogenetic relationships to distinguish paralogues.

### 3.2. Multiple Sequence Alignment

**Figure 1:**
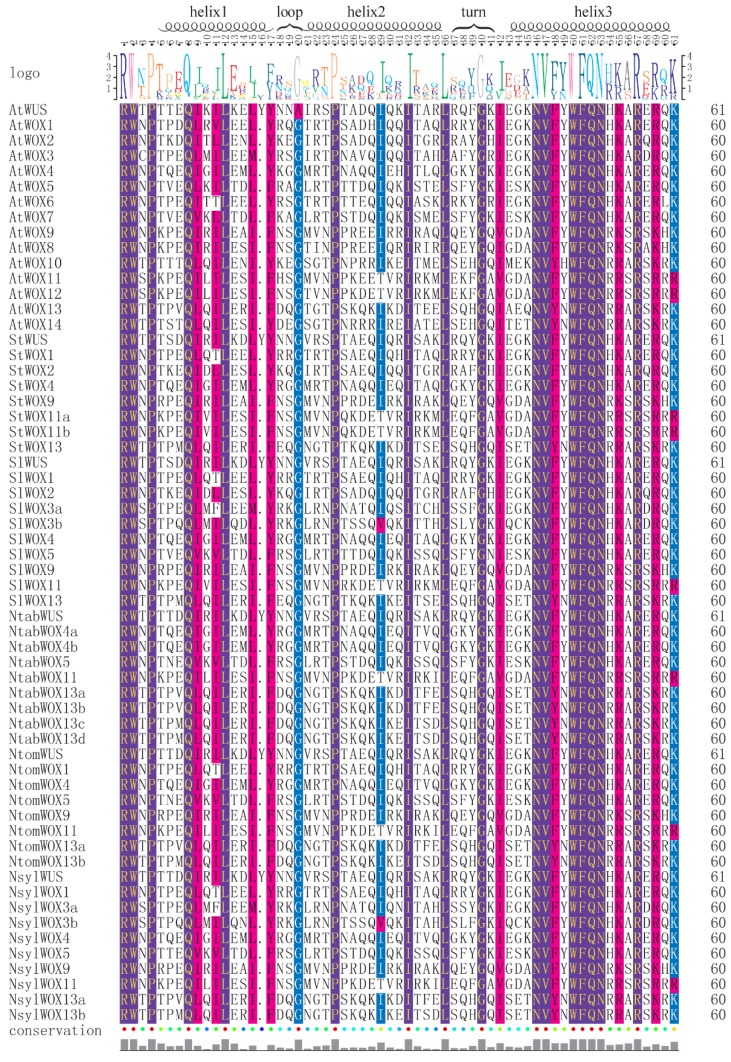
Homeodomain domain sequence analysis of WOX (WUSCHEL-related homeobox) family members. The homeodomain of the newly identified WOX family members contains the typical helix-loop-helix-turn-helix structure. The completely conserved residues are purple colored and highly conserved residues are red or blue colored, all visualized by Texshade (https://ctan.org/pkg/texshade).

To examine features of the homeodomain sequence within the WOX proteins, multiple sequence alignment was carried out with MAFFT using homeodomain sequences of the 60 WOX proteins, including the 45 *Solanaceae* WOXs identified in this study and the 15 *Arabidopsis* WOX family members. The alignment results showed that the homeodomain exhibited a high-level conservation among the six plant species (Figure 1). The typical size of the homeodomain was found to be 60 amino acids with a helix-loop-helix-turn-helix structure. It has been reported that the homeodomain harbors 11 conserved amino acid residues including Q, L and Y in helix 1, I, V, W, F, N, K, R and R in helix 3 [36]. Six more residues were identified to be conserved among homeodomain sequences from rice, sorghum, maize, *Arabidopsis* and poplar, including P, I and L in helix 2, F and Q in helix 3 and G in the turn [3]. Not surprisingly, it was found that all the above mentioned 17 amino acid residues are highly conserved among the 60 homeodomain sequences in this study. In addition, three highly conserved residues were identified from homeodomain sequences of the *Solanaceae* WOXs, including E in helix 1, I in helix 2 and N in helix 3.

### 3.3. Phylogenetic Analysis

**Figure 2:**
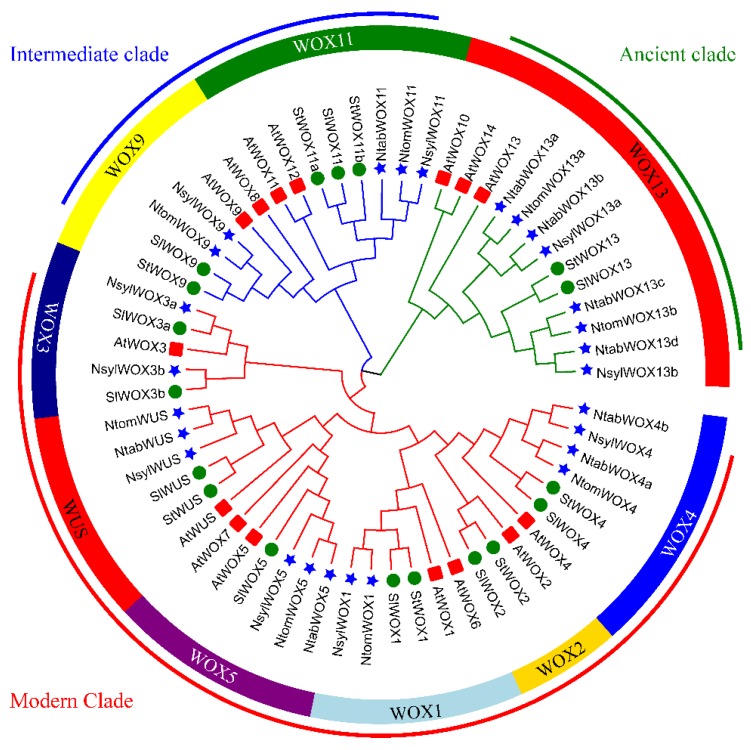
Phylogenetic analysis of WOX family proteins. The phylogenetic tree was generated from the alignment of 60 WOX proteins with 1000 bootstrap replicates. The WOX proteins were classified into three clades and nine subgroups based on phylogenetic analysis using the neighbor-joining (NJ) method. Red squares indicate *Arabidopsis WOX* genes, green circles indicate potato and tomato *WOX* genes, and blue stars indicate tobacco *WOX* genes.

**Figure 3:**
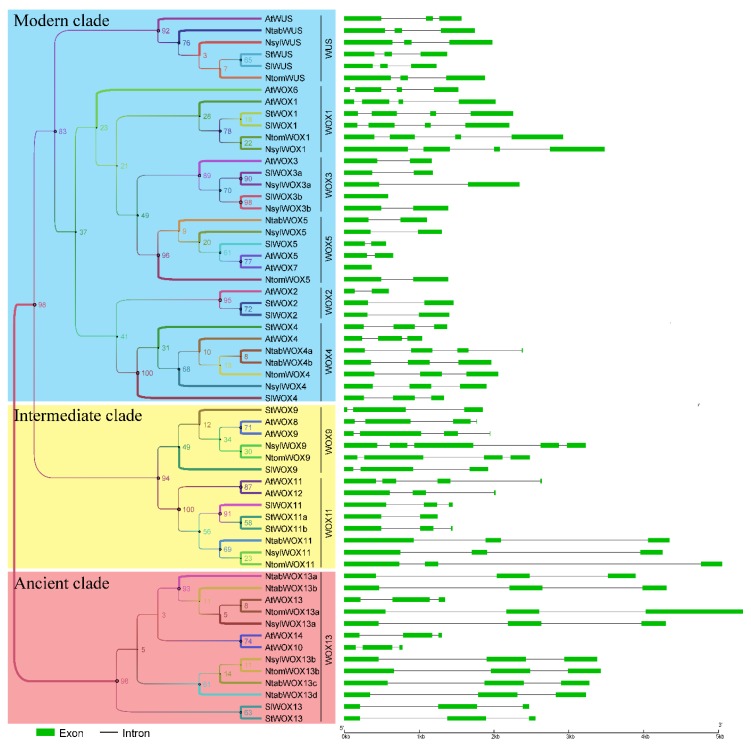
Maximum-likelihood (ML) tree and gene structures of *WOXs* in Arabidopsis, tomato, potato and tobacco. The phylogenetic tree summarizes the relationships within the 60 members of the WOX family. The tree was constructed by PhyML. Exons and introns are represented by green boxes and grey lines, respectively.

**Figure 4:**
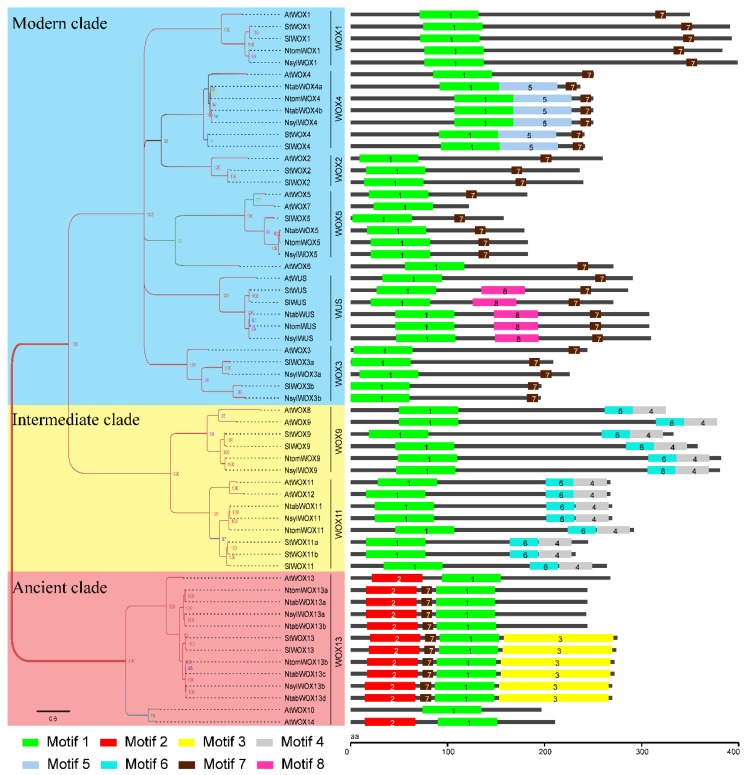
Bayesian inference (BI) tree and motif analysis. The phylogenetic tree revealed the relationships among the 60 members of the WOX protein family, which was generated by MrBayes. The conserved motifs identified by MEME v4.9.1 (http://meme-suite.org/) are highlighted with colored boxes.

To investigate the phylogenetic relationship between WOX members of the six dicotyledonous species and to group them into subfamilies, we performed phylogenetic analysis based on the multiple sequence alignment of full-length or homeodomain sequences of the 60 WOX proteins. To gain more confidence on the reliability of our results, three different methods of tree construction were used, including NJ (Figure 2), ML (Figure 3) and BI (Figure 4). We found good support values with similar topologies for all three trees. In consistent with previous studies [25,37], WOX members fell into three well-organized clades with 33, 14 and 13 members in the modern clade, the intermediate clade and the ancient clade, respectively.

To get further insight into the evolution process, the 60 WOX proteins were sub-divided into 9 subgroups (Figure 2). The ancient clade contains the WOX13 subgroup with 13 WOX members, harboring AtWOX13, AtWOX10, and AtWOX14. The intermediate clade which contains 14 WOXs homologous to AtWOX8, AtWOX9, AtWOX11 and AtWOX12, was further divided into two subgroups, the WOX9 subgroup and the WOX11/12 subgroup. The modern clade (or the WUS clade) contains 33 WOXs that are homologous to AtWOX1–7 and AtWUS. The members in the modern clade were further divided into 6 subgroups, namely the WOX1, WOX2, WOX3, WOX4, WOX5/7 subgroups and the WUS subgroup.

WOX proteins in each of the three major clades were identified from all the five *Solanaceae* species, confirming that the divergence of the *WOX* gene family occurred before the divergence of the six plant species in this study. However, no homologue of AtWOX6–8, AtWOX10 and AtWOX14 was identified in the *Solanaceae* species, suggesting possible gene duplication of *Arabidopsis* WOX family members. The notion of gene duplication in *Arabidopsis* is also supported by the fact that two pairs of highly homologous WOX proteins (AtWOX11/12 and AtWOX10/14) that share a node on the phylogenetic tree were identified in *Arabidopsis* while no such homologue pair was found in the *Solanaceae* species (Figure 2). As expected, WOX families of the five *Solanaceae* species were found more closely related to each other than to that of *Arabidopsis*. All the close interspecies orthologous pairs that share a node on the phylogenetic tree were pairs between the *Nicotiana* species or between tomato and potato.

The WUS subgroup contained a complete set of orthologous sequences from all the six plant species, implying conserved and specified functions of the WUS subgroup members. No homologue of the WOX2 subgroup was identified in the *Nicotiana* species. No WOX3 subgroup member was identified from potato, *N. tomentosiformis* or *N. tabacum*, implying possible deletion events during evolution. In the WOX4 and WOX13 subgroups, the number of NtabWOXs is twice as many as that of NsylWOXs and NtomWOXs. It is likely that in subgroups WOX4 and WOX13, the allotetraploid *N. tabacum* gained equal number of genes from each of its diploid ancestors.

### 3.4. Exon-Intron Organization

Exon-intron organization could provide useful information about the evolution of a gene family. WOX members within the same subgroup were found to have similar exon/intron structures except that genes in the WOX9 subgroup showed complex exon–intron structures and significant variation in the numbers of introns (Figure 3).

A number of *WOX* genes have exon/intron structures distinct from other members of the same subgroup. For example, *NtabWOX*4a has four exons whereas all the other members of the WOX4 subgroup contain three exons. *SIWOX*3b and *AtWOX7* have a single exon while other members of their subgroups all have two exons. Slight variation in exon/intron length was also observed. *NtomWOX11* contains the longest intron of more than 3 kb and *NtomWOX13a* has an exon of more than 1 kb. Such divergence in number or length of exon/intron might be the result of gain or loss of DNA fragments, chromosome rearrangement or fusion, which could ultimately lead to generation of functionally distinct paralogues. Genes in the more conserved ancient clade all have similar exon/intron structures while the modern clade shows the most variation.

### 3.5. Protein Domain Analysis

In addition to the homeodomain, which is present in all the WOX members, seven more conserved motifs were identified using the MEME online tool. The motifs were named Motif 1–8 with Motif 1 being the homeodomain. Supporting the results of the phylogenetic analysis, one or two motifs were found to be shared by WOX proteins within the same clade in addition to the homeodomain: Motif 7 (the WUS box) is conserved in the modern clade, Motif 6 and 4 are present in all the intermediate clade members but not in the other two clades, and Motif 2 is unique to the WOX members of the ancient clade (Figure 4; Supplementary Dataset S3).

A number of protein motifs were identified in this study which might be specific to the *Solanaceae* family or related species. This includes Motif 5 in the WOX 4 subgroup and Motif 8 in the WUS subgroup that are present in all the *Solanaceae* WOX proteins but not in AtWOXs. Similarly, Motif 3 is only present in some of the *Solanaceae* WOXs in the ancient clade (Figure 4).

**Figure 5:**
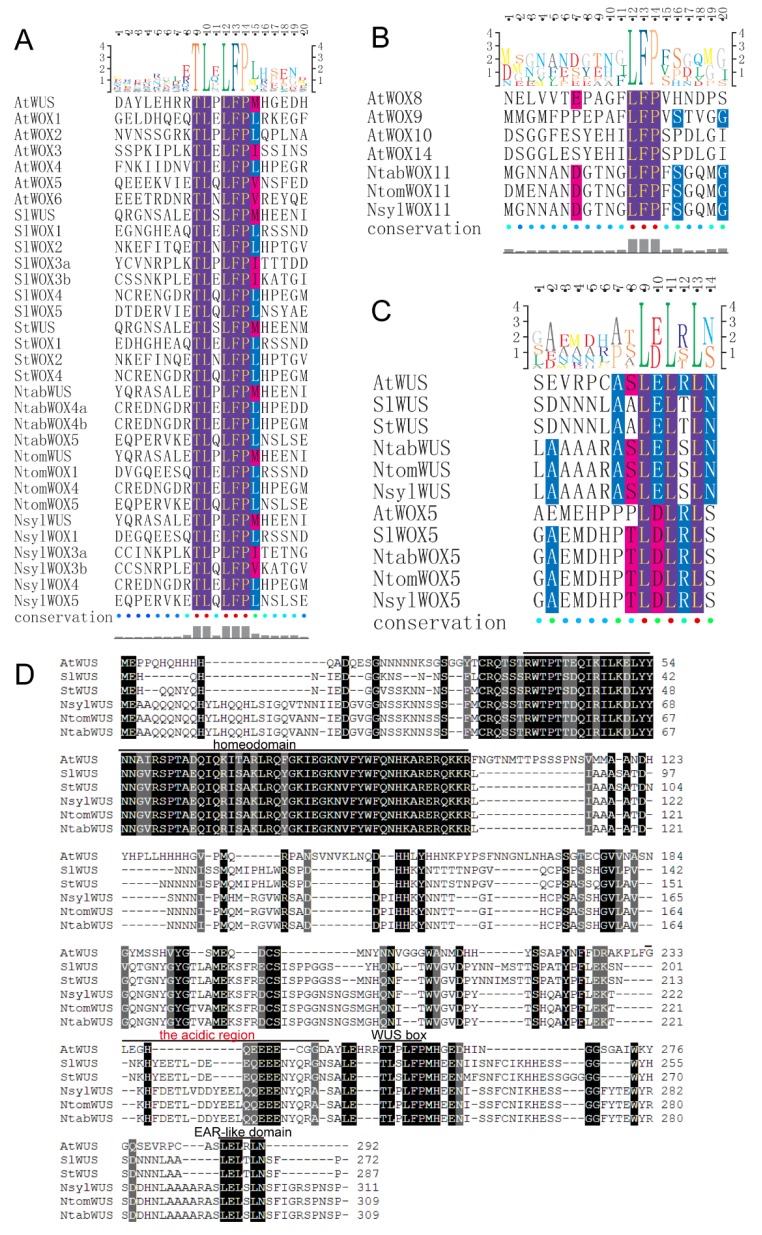
Functional domain analysis of WOX transcription factors in *Arabidopsis*, tomato, potato and tobacco. (**A**) Alignment of the WUS box. 32 WOXs were found to contain the WUS box and all of them belong to the modern clade. The completely conserved residues are purple colored and highly conserved residues are red or blue colored, all visualized by Texshade. (**B**) Alignment of the partial WUS box. The partial WUS box was observed in the WOX11 subgroup of the three tobacco species, containing a TN-LFP motif. The completely conserved residues are purple colored and highly conserved residues are red or blue colored, all visualized by Texshade. (**C**) Alignment of the EAR-like motif. All the identified WOX members in the subgroups WUS and WOX5 contain this functional domain. Conserved residues are shown below the alignment. The completely conserved residues are purple colored and highly conserved residues are red or blue colored, all visualized by Texshade. (**D**) Alignment of the acidic region. SlWUS, StWUS, NtabWUS, NsylWUS and NtomWUS were aligned by MAFFT. The completely conserved residues are black box shaded and highly conserved residues are grey box shaded.

It has been reported that three functional domains of AtWUS, including the acidic region, the WUS box and the EAR-like motif, contribute significantly to its function as a transcription factor [2,38,39]. Among these functional domains, the WUS box domain is only present in members of the modern clade. In this study, a WUS box was identified in 32 of the 33 modern clade WOX members (Figure 5A). Interestingly, a partial WUS box was identified in the WOX11 subgroup members of the intermediate clade in the *Nicotiana* species. The partial WUS box contains a TN-LFP motif instead of the TL-LFP motif as in the WUS box (Figure 5B). The conservation of this partial WUS box in the *Nicotiana* species might be related to certain functions of these *WOX* genes and this might be an indication of ongoing evolution of the WUS box. Furthermore, the EAR-like domain was found to be present in all members of the WUS and WOX5 subgroups except AtWOX7 (Figure 5C). Note that AtWOX7 also lacks the WUS box and is the only WOX protein in this study that contains no other conserved motif except the homeodomain. The functional acidic region of AtWUS which is localized at the N-terminal side of the WUS box was found not strictly conserved among the WUS members from the *Solanaceae* species, although all of these proteins contain a (Q/E) Q/E]EEE motif (Figure 5D). According to a previous report [3], the acidic region was not detected in rice and maize, indicating that this functional domain is perhaps only present in WUS subgroup members of certain plants. Interestingly, amino acid Y in helix 1 of the homeodomain was found to be conserved among all the WUS subgroup members (Figure 1).

### 3.6. Expression of WOX Genes in Different Tissues

**Figure 6:**
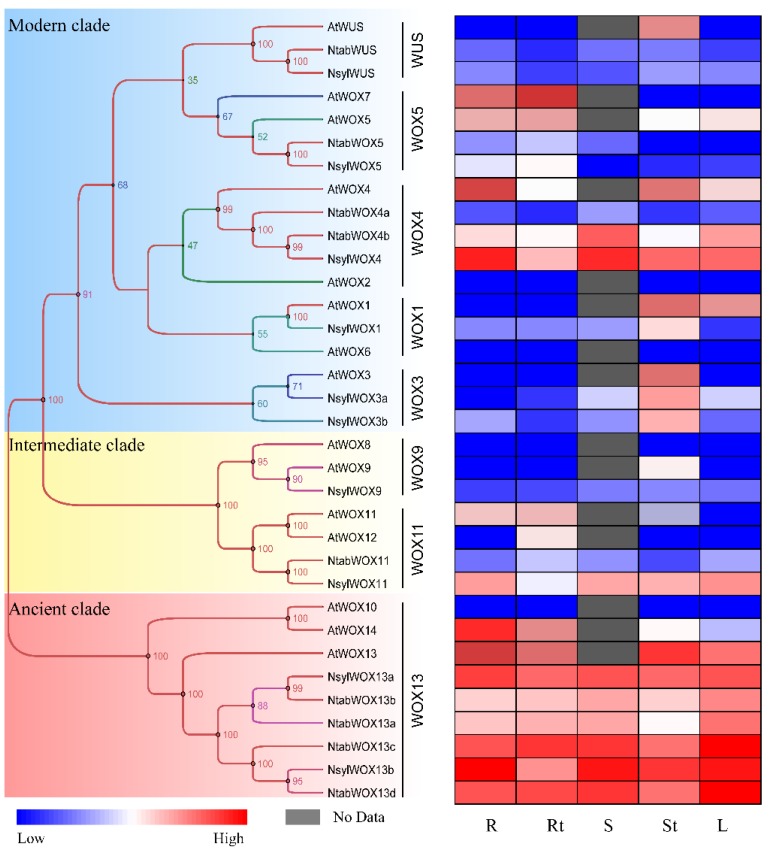
Expression patterns of the WOX gene family members in *Arabidopsis*, *Nicotiana tabacum* and *Nicotiana sylvestris.* The phylogenetic tree was generated by MAGE v.6.06 with 34 WOX members from *Arabidopsis*, *N. tabacum* and *N. sylvestris*. The expression pattern data were normalized and visualized by R. R, root, Rt, root tip, S, stem, St, shoot tip, L, leaf.

In order to get some idea of where the *WOX* genes function in the plant, we analyzed the expression patterns of *WOX* members from Arabidopsis, *N. tabacum* and *N. sylvestris* in different tissue types including root, root tip, stem, shoot tip and leaf at the early developmental stage. Transcript levels of *N. tabacum* and *N. sylvestris* were retrieved from qRT-PCR data. Expression data for *AtWOXs* were collected from the AtGenExpress Visualization Tool. The *WOX* genes were found to be expressed broadly in different tissues with considerable variations between clades, subgroups, and individual genes (Figure 6).

Genes in the same subgroup tended to have similar patterns of expression (Figure 6). Members of the ancient clade, homologues of *AtWOX13* from *N. tabacum* and *N. sylvestris*, including *NsylWOX13a*, *NsylWOX13b*, and *NtabWOX13a–d*, showed a similar expression pattern with relatively high expression levels in most of the five tested tissues. Members of the WUS subgroup exhibited higher expression in shoot tips than in the other tissues, implying that these *WOX* genes might be involved in regulation of shoot apex development like AtWUS. In contrast, members in the WOX5 subgroup were found to be abundantly expressed in roots and root tips, indicating a possible role of NslyWOX5 and NtabWOX5 in root development. Consistent with the role of WOX1 homologues in leaf blade outgrowth and vascular patterning [31,40], *NsylWOX1* showed increased expression at shoot tips and in leaves. *AtWOX3* showed functional redundancy with *AtWOX1* at early stages of leaf development in *Arabidopsis* [41]. It is not surprising that *NsylWOX3a* and *NsylWOX3b* showed a similar expression pattern with *NsylWOX1*. *NtabWOX11* was found to be highly expressed in root tips, suggesting that this gene might have a similar role in root development as its *Arabidopsis* orthologue *AtWOX11* [42].

### 3.7. Expression Changes of Tobacco WOXs upon CLE Peptide Treatments

**Figure 7:**
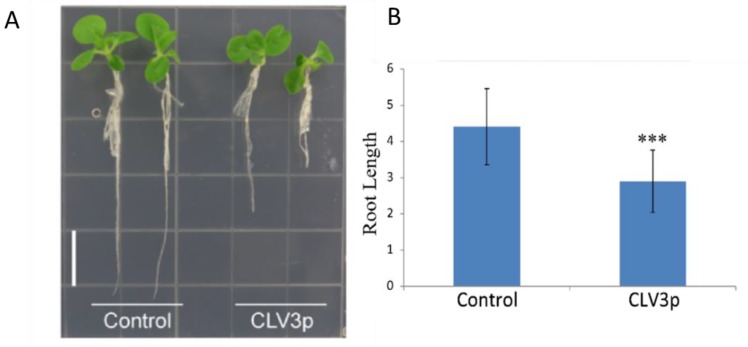
The phenotypes of common tobacco seedlings upon treatments of CLV3p peptides. The peptide treatment experiment was independently repeated for three times, each consisting five individual plants from control as well from CLV3p treated plants. The error bars indicated standard deviation (SD), and asterisks indicated the significant difference compared with the control group (*** *p* < 0.001), calculated by performing *t*-test with GraphPad Prism 5. (**A**) The phenotypes of tobacco growth inhibition upon application of CLV3p. (**B**) Effect of 10 μM CLV3p on root length of tobacco.

**Figure 8:**
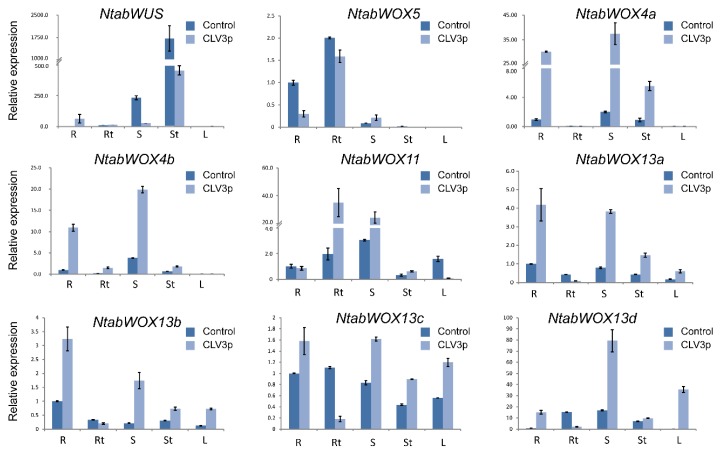
CLV3p-induced changes in transcript levels of *NtabWOXs*. Quantitative real-time PCR was used to detect *NtabWOXs* expression levels treated with CLV3p. These qPCR data presented were obtained from three independent biological replicates with three technical repeats. The error bars indicated SD. R, root, Rt, root tip, S, stem, St, shoot tip, L, leaf.

To understand potential functions of the WOX members in CLE peptide signaling, we treated tobacco plants with synthesized CLV3p and changes in expression of the nine *N. tabacum WOX*s after peptide treatments were determined. As expected, treatments with 10 μM CLV3p dramatically inhibited the tobacco root growth of tobacco (Figure 7). Three-week-old seedlings were harvested and real-time qPCR was performed with RNA samples extracted from roots, root tips, shoots, shoot tips and leaves. The expression of *NtabWOX5* in root and root tip was significantly lower in the CLV3p treated seedlings. Similarly, *NtabWUS* expression in shoot and shoot tips was significantly inhibited by CLV3p treatments (Figure 8). In contrast, expression of *NtabWOX4a*, *NtabWOX4b* and *NtabWOX11* was strongly induced by CLV3p treatments. Interestingly, expression of *WOX13a*, *WOX13b*, *WOX13c* and *WOX13d* was promoted in the root, leaf, shoot and shoot tip while significantly inhibited in root tip by CLV3p treatments (Figure 8). Overall, *NtabWOXs* showed transcriptional responses to CLV3p treatments, suggesting their potential roles in various developmental processes mediated by CLE peptide signaling.

### 3.8. Subcellular Localization and Transactivation Activity Assay

**Figure 9:**
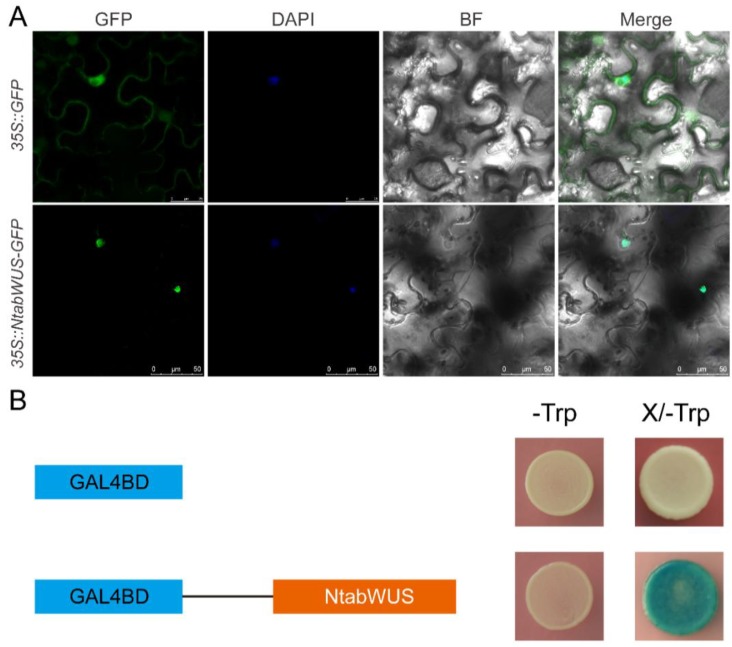
Subcellular localization and transactivation activity assay. (**A**) *NtabWUS-GFP* fusion constructs and green fluorescent protein *(GFP)* driven by the 35S promoter were transiently expressed in tobacco. DAPI (dye 4,6-diamidino-2-phenylindole) staining showed the nucleus. (**B**) *NtabWUS* was fused with the GAL4 (BD) DNA-binding domain in pBridge and transformed into yeast strain AH109. The transformed yeast cells were streaked on the SD/−Trp. BF, bright field; Trp, tryptophan.

To explore potential roles of the newly identified WOX members as transcription factors, the subcellular localization of NtabWUS was investigated. The ORF of *NtabWUS* excluding the stop codon was cloned to be in frame with a GFP reporter gene, which was under the control of the CaMV-35S promoter. The fluorescence of the fusion protein was specifically localized in the nucleus as being verified by DAPI staining, whereas the control signal of *35S:GFP* was distributed throughout the cell (Figure 9A).

In this study, the transactivation activity assay was used to investigate whether NtabWUS has transactivation activity and functions as a transcription factor in regulating the expression of downstream target genes. The coding region of *NtabWUS* was fused with the GAL4 DNA binding domain (GAL4 BD) in pBridge and transformed into yeast strain AH109, which is an engineered strain with UAS fused with reporter genes. The transactivation activity was evaluated based on reporter gene activity. The yeasts were selected on SD medium lacking tryptophan (SD/−Trp). Then, the yeast transformants with the *pBD-NtabWUS* construct turned blue in the presence of X-β-Gal, whereas the negative control did not (Figure 9B), indicating transactivation activity. Taken together, these results indicate that NtabWUS may function as a transcriptional activator.

### 3.9. Genetic Complementation Analysis

**Figure 10:**
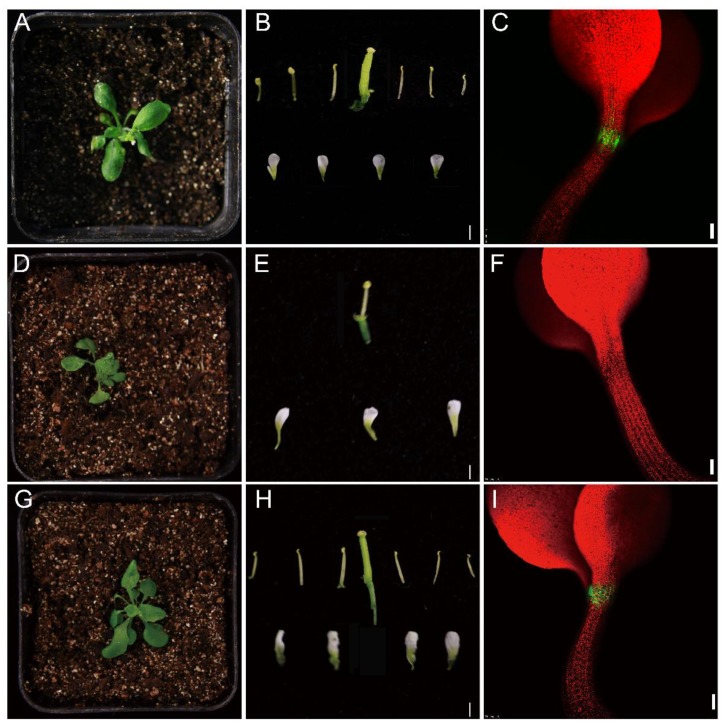
Analysis of NtabWUS function in maintenance of shoot apical stem-cell niche and floral development. Contrast to the wild-type (**A**,**B**), the *wus-1* had defective shoot meristem and flower development (**D**,**E**). J2341, the GFP enhancer trap line, showed specific GFP expression in the shoot apical meristem (SAM) of wild-type *Arabidopsis* shoots (**C**), but not in the *wus-1* mutant (**F**). (**G**–**I**) Interspecies rescue experiments with 4 lines with *NtabWUS* expressed in *Arabidopsis wus-1* plants showed that NtabWUS can rescue the defects of *wus-1* in *Arabidopsis*. Scale bars: 1 mm (B, E, H) and 100 μm (C, F, I).

To further investigate potential functions of the identified *WOX* genes, we tested *NtabWUS* for its ability to rescue the *Arabidopsis wus-1* mutant, which carries a loss-of-function point mutation in the gene *AtWUS*. Compared to wild-type, the *wus-1* mutant had a clearly defective shoot meristem [5,43] and failed to develop into a normal inflorescence (Figure 10A,D). Additionally, in contrast to the wild-type, *wus-1* plants showed severe defects in floral organ development. The wild-type flowers contained six stamens and one central gynoecium (Figure 10B), while *wus-1* flowers had only one central stamen present with no central gynoecium (Figure 10E). Besides, the GFP of the enhancer trap line J2341, which showed specific expression in the SAM [44] and in the distal meristem of wild-type roots [45], was absent in the *wus-1* mutant (Figure 10C,F), confirming the disordered developmental process of the SAM in *wus-1*.

In previous studies, genetic complementation experiments showed that the expression of *AtWOX5* and *AtWUS* driven by the native WUS promoter rescued both premature termination of the SAM and the floral organ developmental defects in *wus-1*. While the expression of the *WUS* lineage *AtWOX4* failed to rescue the *wus-1* phenotypes. AtWOX9 and AtWOX13 were also unable to rescue the *wus-1* SAM defects. These results indicate that the ability to maintain the *Arabidopsis* shoot stem-cell niche is not a common functional character of all the AtWOX family members [14].

NtabWUS contains the conserved homeodomain and the WUS/EAR motifs. When driven by the *Arabidopsis AtWUS* promoter, expression of *NtabWUS* was able to rescue the *Arabidopsis wus-1* mutant phenotypes to wild-type. The *AtWUS:NtabWUS* plants had normal floral organs and J2341 signal (Figure 10G–I), implying that NtabWUS might also function in maintaining the shoot stem cell niche in tobacco.

## 4. Discussion

Forty-five *WOX* genes from five *Solanaceae* species were identified and analyzed. Phylogenetic analysis with three different methods revealed similar topologies and these results were further supported by exon-intron organization analysis, motif analysis and expression analysis. The *Solanaceae* WOX members together with 15 *Arabidopsis* WOX proteins fell into three well-organized clades and were further divided into nine subgroups. Together with the results from expression analysis, peptide treatments, subcellular localization, transactivation activity, and complementation of known mutants, results from this study suggest that the *WOX* genes in the *Solanaceae* species are highly conserved in structure as well as functions in peptide signaling. Unique features in some of the WOX members are however also evident partially due to the extensive expansion of some subgroups through gene duplication as well as gene loss in some other subgroups after species divergence.

In the ancient clade, an expansion event was observed in the WOX13 subgroup in the three *Nicotiana* species. Four WOX13 subgroup members from the *Nicotiana* species, NtabWOX13c, NtabWOX13d, NsylWOX13b and NtomWOX13b were clustered together with StWOX13 and SlWOX13, members from potato and tomato respectively. The other four subgroup members from tobacco, NtabWOX13a, NtabWOX13b, NsylWOX13a and NtomWOX13a, however, were found to form a separate branch on the phylogenetic tree (Figure 2). The isolated group of WOX13 members were also found different in exon-intron organization and protein motifs. In comparison with the original members, these isolated members were found to have larger gene size with longer introns (Figure 3). Furthermore, the isolated members did not contain Motif 3 which was found to be present in all the other WOX13 subgroup members from the *Solanaceae* species (Figure 4). Within the same plant species distinct patterns of expression were observed between the original members and isolated members (Figure 6). The isolated members might be the result of gene duplication after the separation of the *Nicotiana* species from other *Solanaceae* plants.

The WOX9 and WOX11 subgroups that form the intermediate clade exhibited a highly conserved motif organization (Figure 4). The gene structures of these two subgroups, however, showed the highest variation compared with subgroups in the ancient clade and the modern clade, with exon numbers ranging from 3 to 5 within the genes of WOX9 subgroup and 2–4 within the WOX11 subgroup genes (Figure 3). Unlike WOX members of the other two clades, where genes in the same subgroup generally showed similar expression patterns, genes in the WOX9 and WOX11 subgroups were found to have distinct patterns of expression (Figure 6). All these suggested that the intermediate clade might be a rapidly evolving clade with its members showing large variations in gene structures, expression patterns and potential gene functions. In consistent with this hypothesis, distinct functions of WOX9 proteins from *Arabidopsis* and *Petunia*, and other *Solanaceae* species have been reported. The *Petunia* EVERGREEN/WOX9 has been shown to be essential for inflorescence development and architecture [46] while *Arabidopsis* WOX9/STIP together with WOX8/STPL is required for embryo patterning and vegetative SAM maintenance [22].

All WOX members of the modern clade contain a homeodomain and a WUS box except AtWOX7 in *Arabidopsis*. In comparison with its close homologue AtWOX5 and other members of the WOX5 subgroup, AtWOX7 lacks the second exon which harbors the WUS box (Figure 3, Figure 5). The WUS box has been shown to be the functional domain of AtWUS required for induction and maintenance of SAM cell identity. Although evolutionary closely related to AtWOX5 (Figure 2), lack of the critical WUS box may make the function of AtWOX7 different. Other than AtWOX7, which lacks homologue in the *Solanaceae* species, the motif organization and gene structures, as well as expression patterns of the modern clade members within the same subgroup were found to be similar.

In this study, significant difference was observed in expression patterns of *WOX* genes in different modern clade subgroups (Figure 6). Preferred expression of *NtabWUS* in shoot tips and *NtWOX5* in root tips implies conserved functions of these two genes in the *Nicotiana* species. Differential expression changes of these genes in tested tissues upon CLE peptide treatments suggested the involvement of these WOX genes in peptide signaling that regulates various developmental processes (Figure 8). Particularly, the expression of *NtabWUS* could be suppressed by CLV3 peptide treatments, suggesting that CLV3 signaling also limits *WUS* expression in tobacco. In *Arabidopsis*, while CLV3 signaling limits expression of *WUS*, transcriptional factor WUS could promote the *CLV3* transcription to form a feedback loop. NtabWUS subsequently was found to be localized in the nucleus and possessed transcriptional activation activity, suggesting that NtabWUS can activate *CLV3* expression in tobacco and form a similar feedback loop. Further, genetic complementation analysis was carried out and it was found that expression of *NtabWUS* can rescue the *Arabidopsis wus-1* mutant phenotypes. These results suggest that NtabWUS has conserved functions in the CLV3 peptide signaling pathway in maintaining shoot apical stem cell activity in tobacco.

## Supplementary Materials

The following are available online at http://www.mdpi.com/2073-4425/9/5/260/s1. Supplementary data associated with this article can be found in the online version. Supplementary Table S1. qRT-PCR primers used in this study. Supplementary Dataset S1. Protein sequences of the *WOX* gene family members in this study. Supplementary Dataset S2. The multiple sequence alignment of the full-length protein sequences of the *WOX* gene family members in this study using MAFFT. Supplementary Dataset S3. Schematic illustration of motifs in the WOX family members. The motif characterization was based on full-length proteins by MEME.
