# Correction: Li, X.; et al. Identification and Characterization of the WOX Family Genes in Five *Solanaceae* Species Reveal Their Conserved Roles in Peptide Signaling. *Genes* 2018, *9*, 260.

**DOI:** 10.3390/genes9090457

**Published:** 2018-09-13

**Authors:** Xiaoxu Li, Madiha Hamyat, Cheng Liu, Salman Ahmad, Xiaoming Gao, Cun Guo, Yuanying Wang, Yongfeng Guo

**Affiliations:** Key Laboratory for Tobacco Gene Resources, Tobacco Research Institute, Chinese Academy of Agricultural Sciences, Qingdao 266101, China; moxuanpds@163.com (X.L.); star_143_101@hotmail.com (M.H.); lc4115225111@163.com (C.L.); safitfa@yahoo.com (S.A.); gaoxiaoming@caas.cn (X.G.); guocun_zzu@163.com (C.G.)

The authors wish to make the following change in their paper [1]. Due to an undetected mistake, one of the author’s name was written incorrectly. The name of Ahmad Salman is here corrected to Salman Ahmad.

The manuscript will be updated and the original will remain online on the article webpage, with a reference to this Correction.
